# Data collection on the use of embryo bioassays with aquatic animals for toxicity testing and hazard assessment of emerging pollutants

**DOI:** 10.1016/j.dib.2020.105220

**Published:** 2020-01-31

**Authors:** Ricardo Capela, Jeanne Garric, Luís Filipe Costa Castro, Miguel Machado Santos

**Affiliations:** a CIMAR/CIIMAR – Interdisciplinary Centre for Marine and Environmental Research, Av. General Norton de Matos s/n, 4450-208 Matosinhos, Portugal; b FCUP – Faculty of Sciences of the University of Porto, Rua do Campo Alegre s/n, 4169-007 Porto, Portugal; c IRSTEA - National Research Institute of Science and Technology for Environment and Agriculture - Centre de Lyon-Villeurbanne, 5 rue de la Doua, CS20244, 69625 Villeurbanne Cedex, Lyon-Villeurbanne, France

**Keywords:** Aquatic metazoans, Embryo-tests, OECD, Testing guidelines, Legislation, Prioritization tests, Screening tools

## Abstract

A thorough bibliographic survey on the use of embryo-tests with aquatic animals for toxicity testing was performed. The data regarding to the compounds sensitivity (NOEC, LOEC, EC50 and LC50), the available resources for the different animal models (knowledge on the life-cycle, amenability for laboratory breeding, number of embryos produced and reproductive strategy, genomic and transcriptomic resources), together with the European pieces of legislation regarding to animal testing and the available testing guidelines of national and international agencies (OECD, EPA, ISO, ASTM, ICES) were gathered, aiming to the standardization of new embryo-test model species for toxicity testing of new and existing compounds. The data contained in this Data in Brief article is presented and discussed in the review article with the title Embryo bioassays with aquatic animals for toxicity testing and hazard assessment of emerging pollutants: a review [1]. The dataset is provided with this article as a supplementary file.

**Table:** Specifications Table

Subject	Environmental Science: Management, Monitoring, Policy and Law
Specific subject area	Ecotoxicology, Hazard assessment
Type of data	Tables
How data were acquired	Bibliographic Survey
Data format	Raw
Parameters for data collection	Information on the use of embryos, larvae or juvenile aquatic animals on toxicity testing was gathered. NOEC, LOEC, EC50 and LC50s were recorded. The available resources by model, available legislation and guidelines were also gathered.
Description of data collection	A thorough bibliographic survey was preformed. Only research papers published in indexed journals were considered for this collection.
Data source location	Institution: CIIMAR – Interdisciplinary Centre for Marine and Environmental Research City/Region: Matosinhos/Porto Country: Portugal
Data accessibility	With the article
Related research article	Author's name: Ricardo Capela, Jeanne Garric, Luís Filipe Costa Castro, Miguel Machado Santos Title: Embryo bioassays with aquatic animals for toxicity testing and hazard assessment of emerging pollutants: a review Journal: Science of Total Environment DOI: https://doi.org/10.1016/j.scitotenv.2019.135740

**Table:** 

**Value of the Data** • This data gathers information about the use of embryo-tests with aquatic animals for toxicity testing, including the characteristics and available resources for the different animal models, existing guidelines, legislation and compounds sensitivity, which can guide other researchers towards the optimization and development of existing and new embryo-test methodologies. • Both researchers and agencies working to improve the toxicity testing methodologies can use this data in order to standardize or even develop new methodologies based on embryo-tests, fitting within the current legal requirements. • The gathered data can be used to compare the sensitivity between the current methodological approaches, providing the sensitivity data regarding the embryo-test methodologies of several models to different compounds/compound classes. • This data collection gathers a vast array of disperse information regarding the use of embryo-tests for toxicity testing together with the available models, recourses and related information, that may allow to identify the current gaps and to prioritize future research approches regarding the field.

## 1 Data

The data collected consists of four excel tables that are provided as a supplementary file to this publication (Data in Brief dataset). The Annex 1 (Resources by species), gathers the available information for each model species and taxa: Guidelines, Life-cycle, Laboratory cultures, Number of embryos and embryo transparency and Genomic and Transcriptomic resources. The Annex 2 (Testing guidelines), compiles the available testing guidelines by model species developed by National and International Agencies (OECD, EPA, ISO, ICES, ASTM). The Annex 3 (European regulations) gathers the current and previous European legislations regarding to animal experimentation and hazard assessment. The Annex 4 (Sensitivity data), gathers the sensitivity data obtained in embryo, and larval tests, with aquatic metazoans for different contaminant classes.

## 2 Experimental design, materials, and methods

A thorough bibliographic survey on the use of embryo-tests with aquatic animals for toxicity testing was preformed. Information on the use of embryos, larvae or juvenile aquatic animals on toxicity testing was gathered. NOEC, LOEC, EC50 and LC50s for the different model species using different testing compounds were collected, together with the assay characteristics (duration, assay type and endpoints). Only research papers published in indexed journals were considered for this collection. Sensitivity data were converted to microgram per liter (μg/L) when necessary and no further processing was applied. The available resources by model (bibliographic survey), available legislation (https://eur-lex.europa.eu/homepage.html) and guidelines (OECD, ISO, EPA, ASTM and ICES were considered) were also gathered. The collected information is presented and discussed in a review paper [1].
